# Laccase and dye-decolorizing peroxidase-modified lignin incorporated with keratin-based biodegradable film: An elucidation of structural characterization, antibacterial and antioxidant properties

**DOI:** 10.1016/j.fochx.2023.101035

**Published:** 2023-11-24

**Authors:** Syed Waqas Ali Shah, Keyu Ma, Riaz Ullah, Essam A. Ali, Abdul Qayum, Zahoor, Nisar Uddin, Daochen Zhu

**Affiliations:** a Biofuels Institute, School of Emergency Management, School of the Environment and Safety Engineering, Jiangsu University, Zhenjiang 212013, China; b School of Food and Biological Engineering, Jiangsu University, 301 Xuefu Road, Zhenjiang, Jiangsu 212013, China; c Department of Pharmacognosy, College of Pharmacy, King Saud University, Riyadh, Saudi Arabia; d Department of Pharmaceutical Chemistry College of Pharmacy, King Saud University, Riyadh, Saudi Arabia

**Keywords:** Alkali-lignin, Keratin, Laccase enzyme, Dye-decolorizing peroxidase enzyme, Sustainable films

## Abstract

Lignin valorization to produce functionalized materials is challenging. This study harnessed the versatile properties of lignin through a grafting reaction involving the aryl hydroxyl group of alkali lignin (AL) and enzymatically modified-alkali lignin (EMAL) using *Bacillus ligninphilus*-derived laccase (Lacc) L_1_ and *C. seriivinvornas*-derived dye-decolorizing peroxidase (DyP) with keratin (K) amide group. This reaction was executed utilizing an eco-friendly solvent with the aim of generating thin films. A thorough investigation was conducted, focusing on grafting AL and EMAL onto K. The incorporation of EMAL into the films enhanced tensile strength (TS) (14.8±1.8 MPa) and elongation at break (EAB) (23.7±0.3 %). Additionally, it enhanced thermal stability, suppressed the proliferation of *Staphylococcus aureus* (*S*. *aureus)* and *Escherichia coli (E. coli)*, and mitigated oxidative stress. This study introduces a novel approach for lignin valorization, offering the potential to tailor mechanical properties, antibacterial and antioxidant properties of the final material, making it sustainable substitute for petroleum-based products.

## Highlights

• Enzymatic modification of lignin by laccase and dye-decolorizing peroxidase.
• Development of ecofriendly films from animal and plant biomass.
• Biodegradable lignin-based films substitute for petroleum-based plastics.

## Introduction

Lignin an aromatic biopolymer, less-expensive by-product of paper and cellulose industries and ethanol-biorefineries (Bajwa et al., 2019). Molecular structural features (methoxyl- and hydroxyl-substituted phenylpropane units (guaiacyl, syringyl, and *p*-hydroxyphenyl) give it amphiphilicity and unique bio-activity, bio-compatibility, antimicrobial, antioxidant, and UV-shielding properties (Jiang et al., 2023, Shah et al., 2023). Due to rich in phenolic compounds, lignin can be used in various polymers and biomaterials production (Chio et al., 2019). Though, lignin low reactive nature and complex structure limit its further applications, and most of the lignin is burned as a fuel or discarded as a waste. Therefore, lignin depolymerization is crucial and challenging for its utilization in value added materials production (Chio et al., 2019). Lignin depolymerization/partial degradation can be achieved *via* biological, chemical, and mechanical methods. In spite of various physio-chemical methods, biological depolymerization of lignin and its transformation to value-added-products is considered a cost-effective and environmentally friendly approach (Murillo-Morales et al., 2023, Sethupathy et al., 2023, Zhu et al., 2020).

Microbial-derived extracellular-enzymes, such as laccase (Lacc) and dye-decolorizing peroxidase (DyP) have the potential of lignin depolymerization (Sethupathy et al., 2023, Zhu et al., 2020). Lacc and DyP are the members of oxidase and haem peroxidase, prevalent in bacteria, fungi, actinomycetes, archaea, and metazoa (Reiss et al., 2013, Yoshida and Sugano, 2023). In spite of other microbes, bacterial Laccs and DyPs enhance the lignin depolymerization (Ehibhatiomhan et al., 2023, Moya et al., 2011), and other attractive properties, such as a wider pH range, thermal stability, and tolerance against various inhibitors (Murillo-Morales et al., 2023, Sethupathy et al., 2023, Zhu et al., 2020). Thus, bacterial Laccs and DyPs are promising for enzymatic modification of lignin.

Plastic (packing material) manufacturing represents a major revolution for humankind. Globally, plastic production from petroleum derived compounds estimated to reach approximately 34 billion tons up to 2050 (Winfield, 2019). In addition, processing industries of proteins (slaughter-houses, meat-packing, leather-processing-plants, etc.,) generates keratin (K) extracted wastes in a higher quantity. Both, plastic and K-extracted wastes increases waste-biomass globally. Burning or burying of this bio-waste adversely affect the environment, due to the release of greenhouse gas during burning and burying leads to underground water contamination. Therefore, the conversion of this bio-based waste into value-added-products, such as thin film is considered not only a sustainable approach for removal of this solid waste, but also an attractive alternate way of evolving an effective bio-based material from renewable resources (Oluba et al., 2021). Keratin present in hairs, wool, nails, and feather of birds, mammals, and reptiles, is an organic biodegradable polymer. Due to strong covalent-bonding potential, stable in hydrophobic interactions and 3-dimensional network of macromolecules. Additionally, thermosetting amendment capacity of K, making it favorable for the production of value-added materials. (Ningthoujam et al., 2018). Therefore, this bio-waste (K) could be a promising value-added commodity in biomaterial production (Saha et al., 2019).

The possibilities and challenges of using lignin crosslinking with chitosan, starch, soy protein for the development of thin films have been described (Crouvisier-Urion et al., 2016, Mohammad Zadeh et al., 2019, Shi and Li, 2016). Although, these studies successfully accomplished the goal of film formation. However, there are some issues to overwhelmed. For example, poor machinal stability, higher brittleness, and dark-color of final product, suggesting that till there is a possibility for precision of these issues. Numerous studies have indicated the incorporation of K for improving the physio-chemical and functional properties of bio-based derived thin films (Alashwal et al., 2020, Sharma et al., 2018). To the authors’ best knowledge, there is no study reported on the synthesis of alkali lignin (AL) and K-based thin films. Therefore, the present study aimed to demonstrate that modified/depolymerized AL (*via* a biotechnological approach and using it for thin film forming with K) can significantly enhance the physio-chemical, thermal, and functional abilities of biomaterials.

In this study, *Bacillus ligninphilus*-derived Lacc L_1_ and *C. seriivinvornas*-derived DyP were employed for the modification of AL in the presence of ABTS as a mediator. The investigation explored the impact of Lacc and DyP treatment on AL, referred to as enzymatically modified alkali lignin (EMAL), and subsequently analyzed the resultant films to assess mechanical and barrier properties. Furthermore, the study assessed the influence of the formulated films on proliferation of *Staphylococcus aureus (S. aureus)* and *Escherichia coli (E. coli)*, as well as their capacity of scavenging 2,2-diphenyl-1-picrylhydrazyl (DPPH), for induction of their prospective application as sustainable and efficacious antibacterial and antioxidant biomaterial.

## Materials and methods

### Materials

Alkali lignin (L) (Sigma^TM^, CAS number 8068-05-1), Keratin (K) powder (Sigma^TM^, CAS number), 2,2′azino-bis (3-ethylbenzothiazoline-6-sulfonic acid (ABTS) (Sigma Aldrich, USA), Isopropyl beta-d-thiogalactopyranoside (IPTG) (Sigma Aldrich, USA), kanamycin sulfate purchased from Sigma Aldrich (USA). Glycerol (99 %), sodium chloride (NaCl), copper chloride dihydrate (CuCl_2_·2H_2_O), Tris (hydroxymethyl) ammonia methane, ethylenediamine tetraacetic acid (EDTA) yeast extract, tryptone, and agar powder purchased from Shinopharm chemical reagent Co. Ltd. (Shanghai, China). Shanghai Luwei Microbial SCI.&TECH. CO.LTD (Shanghai, China) provide the bacterial strains, *Staphylococcus aureus* (*S. aureus*) CMCC(B) (26003) and *Escherichia coli (E. coli)* CMCC(B) 44102). *Bacillus ligninphilus* L_1_ DSM 26145^T^, *Escherichia coli* BL_21_ (DE_3_).

### Extraction and purification of Lacc and DyP enzymes

*E. coli* BL_21_(DE_3_) with Lacc gene (pET_28a_^+^lacc) from *Bacillus ligninphilus* L_1_ DSM 26145^T^ and DyP gene (pET_28a_^+^DyP) from C. *seriivinvornas-*DyP (DSM 26136) (NCBI reference sequence NZ_CP210554.1, bp 924) were used for protein expression and purification. The expression of Lacc and DyP proteins was verified according to our group's previous study (Zhu et al., 2020). Briefly, to express Lacc and DyP, individual colonies of *E. coli* (BL_21_ (DE_3_)/pET_28a_^+^lacc) and *E. coli* (BL_21_(DE_3_)/pET_28a_^+^DyP) were introduced into 10–20 mL of Luria-Bertani (LB) media supplemented with kanamycin (50 µg/mL). The cultures were then incubated at 37 °C in a shaking incubator (180–200 rpm) for duration of 5–8 h. Upon attainment of optical density at 600 nm (OD_600_) with the culture range of 0.6–0.8, protein expression was initiated through the induction of 0.5 mM IPTG. After overnight incubation at 20 °C with 180–200 rpm, cells harvesting was performed *via* centrifugation (5000–8000 x g for 5–10 mins at 4 °C). The cellular pellets were reconstituted in a lysis buffer containing 10 Mm Tris-HCL,1mM EDTA, 100 mM NaCl), followed by sonication (30 %, 6 *sec* on and 4 *sec* off) for a duration 10–30 min. Afterward centrifugation at 18000–20000 x g for 25–30 mins was performed to acquire the supernatant. The Lacc and DyP enzymes were purified using Ni-Bestarose fast-flow column per standard protocol. The chromatography column was pre-conditioned with a buffer comprising 20 mM Tris-HCl, 0.5 M NaCl, pH 8.0. Subsequently, the Ni-Bestarose fast-flow column was then loaded with the supernatant. The column was washed with buffer (20 mM Tris-HCL, 0.5 M NaCl, 20 mM imidazole, pH 8.0) for the removal of unbounded and nonspecific proteins and the Lacc and DyP was eluted with elution buffer (20 mM Tris-HCL, 0.5 M NaCl, 300 mM imidazole, pH 8.0). Proteins were purified by an anion exchange chromatography column (Hi-Trap Q-HP column, GE Healthcare, USA). Through, Bradford assay the concentration of Lacc and DyP concentrations were quantified and were stored at −80 °C. The confirmation of protein molecular weight was achieved using sodium dodecyl sulfate–polyacrylamide gel electrophoresis (SDS-PAGE).

### Decolorization/modification of alkali lignin

Alkaline lignin modification/decolorization was performed based on our previous studies (Sethupathy et al., 2023, Zhu et al., 2020). Briefly, AL (0.55 g) was incubated with 10–20 mL of enzyme (Lacc and DyP) at 37 °C with 180–200 rpm for 24–72 h, with the addition of Tris (150–160 mL (60 mM), (pH 7.00±0.1)), ABTS (15–20 mL (10 mM)), and CuCl_2_·2H_2_O (10–15 mL) (10 mM)). Centrifuged at 8000–10000 x g for 15–20 min. The collected supernatant was freeze-dried (Crist, Beta 1–8 LD plus).

### Films preparation

Enzymatically decolorized/modified alkali lignin (EMAL) and K active films were prepared via solvent casting process with suitable concentrations and pH. K 1 % (w/v) was dissolved in deionized water at 90 °C with the incorporations of 0.85 % of NaOH (w/v). The pH of the K solution was adjusted below the isoelectric point. The AL, laccase-modified-lignin (LML), and dye-decolorizing peroxidase-modified lignin (DML) were first solubilized in ethanol 1–2 % (w/v). and solubilized AL, LML, and DML solutions were mixed with a 1:1 ratio. Then glycerol (10–15 % g/g) was introduced by dropping into the mixture continue stirring at 20–25 °C. Finally, petri dishes of polystyrene with a diameter of 13.5 cm were prepared. The film-forming solution of polymers containing glycerol was poured into the petri dishes and was kept for drying (60 °C, 8–12 h). Dried films were shifted to a climatic chamber (WTC, Binder) at 25–40 % relative humidity (RH). Finally, films were stockpiled inside a desiccator at room temperature.

### Mechanical properties of films

#### Mechanical behavior

The texture analyzer (Vienna Court, Lamas Road, Godalming GU7, 1YL, UK) utilized in this study was calibrated with 2–5 kg mass to evaluate the mechanical properties of films. The standard protocol (NF ISO 527-1) was followed with minor modifications: A/TG test prob was fit with a clamping distance of 20–30 mm, the speed of 2 mm/s was set for crosshead, and the length of the initial gauge was 5 cm. A precision cutter was used to prepare the film samples (n = 3) for the observation of tensile strength (TS, MPa) and elongation-at-break (EAB, %) (Crouvisier-Urion et al., 2016, Rashid et al., 2023).

#### Water vapor permeability (WVP) and moisture content (MC)

The films WVP were observed following the standard protocols of ASTM E96-80, 1980 and modified by Debeaufort et al. (Debeaufort et al., 1993). Briefly, an RH gradient (30–100 %) was selected for the WVP procedure. In a climatic chamber with 30 % RH at room temperature, the films permeability towards water vapors were observed. Before the WVP observations, prepared films were tightly placed on vails containing CaCl_2_ 10 g and placed in a desiccator contained KNO_3_ (RH 93 %). Following the attainment of stable state, the calculation of WVP (g/m/s/Pa) was conducted using the Eq. (1).

(1) WVP = Δ_m_/(Δ_t_*Δ_p_*a) *e

In the above equation, the term Δ_m_/Δ_t_ represented the weight of moisture loss per unit time (g/s), a represented the area of the films visible to moisture (9.08×10^−4^), e for the thickness (m) of the films, and Δ_P_ represented the partial pressure of water vapor in the films (2.94 kPa). Three replicates were performed.

The films dried to constant weight were used to calculate the MC using the following equation:

(2) MC (%) = M_1_-M/M_1_ *100 %

#### Color coordinates

The lightness, red-green, and yellowness of the films were evaluated using a color quest XE colorimeter (Reston, Virginia, USA). The color differences were calculated as:

(3) ΔE = [ (L-L*)^2^ + (a-a*)^2^ + (b-b*)^2^]^0.5^

L* stands for lightness, a* for red-green, and b* for blue-yellow values of the measured film samples. Here, L, a, and b represent the standard white plate values.

### Physio-chemical characterization of films

The Fourier transform infrared (FT-IR) spectroscopy of films was conducted using a Nicolet iS50, Thermo electron, USA. The analysis spanned a frequency range of 4000–500 cm^−1^, with a resolution of 4 cm^−1^, employing a germanium crystal attenuated total reflection (ATR) accessory. The thermal analysis of the active films was determined using a thermal analyzer (NETZSCH TG 209 F3, Germany). The samples were heated (30–800 °C) under the nitrogen (N_2_) gas flow (20 mL/min) at 0.3 gas pressure at a rate of 10 °C/min. Differential scanning calorimetry (DSC) (DSC 4000, Perkin, Elmer), was used to determine the heat flow (30–200 °C) under the N_2_ gas flow (20 mL/min) at 0.3 gas pressure at a heating rate of 20 °C min^−1^. SEM (JSM-700IF, JEOL, Ltd., Japan) was used for microstructures of the films. Samples equilibrated at 33 % relative humidity (RH) were afixed onto the conductive platform of the SEM and subjected to a 5 min gold sputtering process. Observation of the film sample was conducted at a mignification of 1000, employing an accelerating voltage of 15 kV under an absolute pressure of 230 Pa. X-ray photon spectroscopic analysis of the films was performed with a PHI Versa-probe 1000 instrument using a monochromatic A1-Kα X-ray. A 100 W A1-Kα-radiation (1486.7 eV) was used as the X-ray source, with the size (200 µm in diameter) of the circulation spot, and with the assimilation of higher resolution (pass energy = 58 eV) 45° angle of emission. Measurements of the samples were performed at room temperature in an ultra-high vacuum compartment with a base pressure of 2.10^−7^. Photoelectron emission with low energy (<10 eV) induces the neutralization of surface charging by electron flood and ion gun. XPS spectra were referenced according to C—C/C—H bonds (carbon 1S line at 284.6 eV) (Crouvisier-Urion et al., 2016). Gaussian-Lorentzian function and XPS peaks were fitted together for deconvolute complex lines.

### Antibacterial properties

The antibacterial characteristics of AL and EMAL with K active films against *S. aureus* CMCC(B) (26003) and *E. coli* CMCC(B) 44102) were assessed using optical density and agar-disc-diffusion methods.

#### Agar disc diffusion method

The antibacterial efficacy of active films against *E. coli* and *S. aureus* was conducted in accordance with established procedures (Alzagameem et al., 2019). Briefly, active films, shaped as a disc with a diameter of 0.5 mm. Subsequently, a fresh culture of *E. coli* and *S. aureus* was evely spread on agar medium plates, and the films discs were placed within the plates, incubated for 24 h at 37 °C. The disc prepared from K films was used as a control.,Ultimately, the zones of inhibition were quantified.

#### Optical density method

The antibacterial efficacy of the films was assessed using the optical density method, as previously described (Rashid et al., 2023; M. Zhang et al., 2023). Briefly, films specimens were fragmented and introduced to tubes containing 10 mL of *E. coli* and *S. aureus* LB media. The tubes were then incubated at 37 °C for 28 h. Optical density measurement at a wave length 560 nm were recorded at interval of 4 h throughout the entire incubation period using a microplate reader (SpectraMax-M2e, Molecular Devices, China). Film composed of K was employed as a control.

### Antioxidant activity

The antioxidant potential of films was assessed through the utilization of the stable free radical 2,2-diphenyl-1-picrylhydrazyl (DPPH). To the 10 mL of DPPH (50 mg/mL) solution in ethanol, approximately 10 mg (10 cm^−2^) of film was introduced. The kinetics of the reaction was measured at the absorbance of 515 nm (WPA light wave UV/visible spectrophotometer) after the DPPH reactant. Prepared film samples antioxidant activity was checked by adding 1–2 mL of the reaction mixture to the measuring vial. After each absorbance, samples were kept in dark under continuous stirring (200 rpm).

The calculation of the in-reduction scavenging activity (%) was performed using the equation (4):

(4) RSA = 100- (a_blank_ – a_sample_/a_blank_) *100

In the above equation, a_blank_ indicated the absorbance associated with DPPH and a_sample_ represented the absorbance associated with film samples. The kinetics were observed at least in triplicate.

### Statistical analysis

The information provided in the tables and figures was expressed in terms of the mean ± standard deviation (SD). Statistical analysis of the data was performed using analysis of variance (ANOVA) *via* Statistical Package for Social Sciences (SPSS) software. To apprise variation in means, Duncan's multiple tests were utilized. A significance level of (*p* < 0.05) was applied to ascertain the significance among the datasets.

## Results and discussion

### Laccase and DyP gene expression and decolorization of alkali lignin

**Fig. 1:**
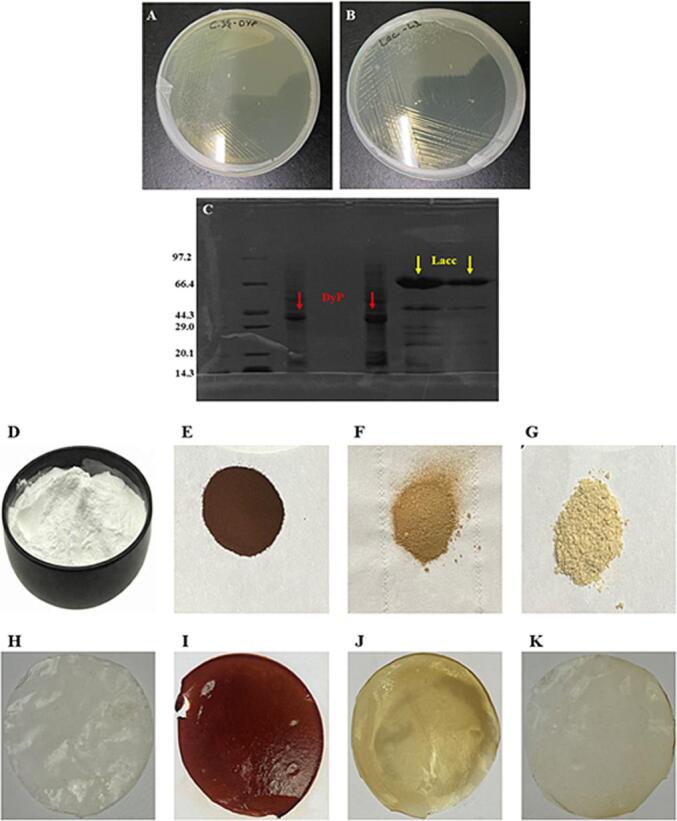
(A-B) *E. coli* BL_21_(DE_3_) plate growth; (C) SDS-PAGE of recombinant laccase and DyP product in *E. coli* BL_21_(DE_3_) cells harboring pET_28a_^+^lacc and pET_28a_^+^DyP; Visual appearance of keratin powder (D), alkali lignin (E), decolorized/modified lignin by laccase (F), and decolorized/modified lignin by DyP (G); visual appearance of keratin film (H), alkali lignin film (I), decolorized/modified lignin by laccase (J), and decolorized/modified lignin by DyP (K).

The recombinant Lacc and DyP genes were cloned into pET_28a_^+^, expressed in *E. coli* BL_21_ (DE_3_), purified by affinity chromatography. Analysis of purified Lacc and DyP showed a band (∼35 DyP) and (∼60 Lacc) on SDS-PAGE (Fig. 1C). The biotechnological approach using Lacc and DyP enzymes significantly decolorized AL (Fig. 1F and G). Previous studies indicated that the separation of kraft lignin into high and low molecular weight fractions induces noticeable changes in their inherent color (Zhang et al., 2017). Briefly, this separation resulted in lighter-color (low molecular-weight fraction) and dark-color (high molecular weight fraction). It is a widely recognized fact that the utilization of lignin imparts a dark-color to composites, which may be perceived as unattractive in certain markets. This dark-color of lignin is due to the presence of aromatic rings containing carboxyl or carbonyl groups, and quinones/quinone methides, methoxyl groups in its structure (Zhang et al., 2017). The decolorization of AL subsequent to the treatment with Lacc and DyP suggests the degradation of AL into aromatic monomer, low molecular weight fractions, and the removal of chemical groups responsible for the lignin’s dark color. The enzymatic degradation of lignin by Lacc and DyP could mitigate the potential concerns in applications where aesthetic appearance is a critical requirement. Moreover, the enzymatic treatment could overcome excessive brittleness and mechanical performances by crystalline arrangement in lignin’s polymeric structure (Murillo-Morales et al., 2023, Sethupathy et al., 2023, Zhu et al., 2020). Over results could provide a new insight for utilizing Lacc and DyP as a promising candidate for lignin depolymerization for producing value-added biomaterials.

### Functional properties of alkali and decolorized lignin’s/keratin films

#### Mechanical properties

The well-known action of Laccs and DyPs on lignin could bring significant deviations in its mass-fraction, low-reactive-nature, mechanical-stability, and thermal properties (Martin et al., 2022, Murillo-Morales et al., 2023, Sethupathy et al., 2023). In this study, the flexibility and strength of AL and EMAL films against external forces were evaluated by TS and EAB. The measured values of TS and EAB for AL and EMAL films are shown in Table 1. Our results of TS (K = 6.5±0.5 MPa, AL = 6.9±1.1 MPa) and EAB (K = 9.2±0.1 %) and AL = 9.8±0.3 %) were found with no significant (*p* < 0.05) differences. However, the addition of LML and DML tends to a comparatively higher increase in the TS and EAB of the films (Table 1). These improved mechanical properties were attributed to the better homogeneity of EMAL in the mixture after enzymatic treatment. Similar observation has been previously reported for improved mechanical properties of thermoplastic and lignin-based composites after enzymatic treatment (Martin et al., 2022, Murillo-Morales et al., 2023, Thao et al., 2022). Lacc and DyP treatment can therefore represents a greener alternative to chemical treatment for improving the mechanical and functional properties of lignin-based materials.

**Table 1:** Tensile Strength (TS), Elongation At Break (EAB), and Thickness of different films previously equilibrated at 33 % RH. Water vapor permeability (WVP), Moisture Content, and Color Coordinates of film samples.

Physical characterization	Film samples				
	K	AL + K		LML + K	DML + K
TS (MPa)	6.5±0.4^a^	6.9±1.1^a^	7.2±0.4^b^		14.8±1.8^c^
EAB (%)	9.2±0.1^a^	9.8±0.3^a^	15.5±0.6^b^		23.7±0.3^c^
Thickness (mm)	0.1±0.0^a^	0.3±0.0^b^	0.3±0.0^b^		0.3±0.0^b^
WVP (10^−10^.g.m^−1^. s^−1^. Pa^−1^)	9.1±1.8^a^	9.0±0.4^a^	6.1±0.8^b^		2.0±0.5^c^
Moisture Content%	47.0±1.5 ^a^	31.2±2.2^b^	19.3±2.7^c^		15.8±1.2 ^d^

Color Coordinates					
L* values	70.8±0.9	31.9±0.9	81.6±0.6		82.97±1.0
a* values	3.1±0.9	4.7±0.7	2.4±0.6		15.1±0.9
b* values	17.0±0.9	9.0±0.8	17.3±0.7		37.1±1.0
ΔE values	19.7±0.7	54.9±0.9	13.1±0.1		49.9±1.1

**Note:** The superscript lowercase letters represents statistical analysis by one-way ANNOVE (n = 3). K (keratin), AL (alkali lignin), LML (laccase modified/decolorized lignin), and DLM (DyP modified/decolorized lignin).

#### Barrier properties

The WVP determines the transfer of moisture from the environment to the product through the internal structure. The WVPs analysis of prepared films (K, AL, LML, and DML) are represented in Table 1. Our results showed no significant (*p* < 0.05) difference for K and AL/K films (K = 9.1±1.8 g.m^−1^. s^−1^. Pa^−1^ and AL + K = 9.0±0.5 g.m^−1^. s^−1^. Pa^−1^). This may be due to the destabilization of the keratin network with the incorporation of lignin, as well as the reduced cohesiveness of the polymers. Previous studies have indicated that lignin addition to gelatin matrix increased the WVP of the final product (Núñez-Flores et al., 2013). However, a significant (*p* < 0.05) decrease was observed in the WVP of films with the incorporation of EMAL (LML + K = 6.1±0.8 g.m^−1^. s^−1^. Pa^−1^ and DML + K = 2.0±0.5 g.m^−1^. s^−1^. Pa^−1^ (T. 2). The reduction in WVP of the EMAL films might be due the low molecular weight fractions and short chains of lignin after enzymatic treatment.

#### Moisture content of lignin’s and keratin films

The MC of the films are shown in Table 1. Our results as compared to K (47.0±1.5 %) film showed a significant (*p*< 0.05) decrease in AL (31.2±2.2), LML + K (19.3±2.7 %) and DML + K (15.9±1.2 %). This could be due to the waterproofing ability of lignin, the smaller mass of EMAL, and improved solubility (Murillo-Morales et al., 2023, Notley and Norgren, 2010). As well, the interaction between biopolymers and hydrophobic proteins reduces the moisture content by limiting the H-bonding of polymers with water molecules.

#### Color measurement

The most important optical property of biopolymer packing films is color. Lignin implies a very dark-color, actually un-attractive. The visual-appearance of the films are represented in Fig. 1H and K. Keratin film is colorless and transparent, by adding lignin, the values of L* (lightness) and b* (yellowness) were significantly (*p* < 0.05) decreased. However, the a* (redness) value significantly (*p* < 0.05) increased (Table 1). These variations in color coordinates indicated the impact of lignin dark-color on the developed film. Lignin dark-color arises from the presence of carboxyl or carbonyl groups, and quinones/quinone methides, methoxyl groups in its structure (Song and Othman, 2022, Zhang et al., 2017). The addition of LML (Fig. 1J) into the film matrix, significantly (*p* < 0.05) increased the color parameters L* (lightness), and b* (yellowness) of LML + K compared to K film (Table 1). However, the a* (redness) value slightly decreased. In the visual appearance of LML film, more yellowness has appeared (Fig. 1J). DLM incorporated film L* (lightness), b* (yellowness) and a* (redness) values were significantly increased (*p* < 0.05) (Table 1). These variation in color coordinates, indicates the elimination of carboxyl or carbonyl groups, and quinones/quinone methides, methoxyl groups (lignin’s dark-color) after Lacc and DyP treatment, might overwhelmed the possible denial in applications where the aesthetical appearance is crucial requirement.

### Molecular interaction of films revealed by FT-IR spectrophotometry

**Fig. 2:**
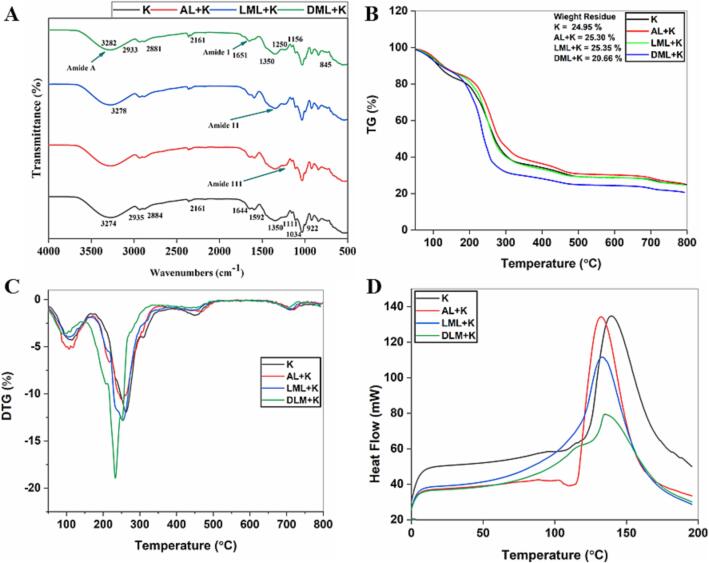
FTIR and thermal stability analysis. (A) Molecular interaction of films by FT-IR spectroscopy. Thermal stability analyses of films (B-D) conducted through thermogravimetric analysis (B); Derivative thermogravimetry analysis (C); and Differential scanning calorimetry analysis (D). K (keratin), AL (alkali lignin), LML (laccase modified lignin), and DML (DyP modified lignin).

The analysis of AL and EMAL films by FT-IR spectrophotometry provides molecular interactions and their confirmation. The prepared films FT-IR spectrum are displayed in Fig. 2A. Briefly, the observed peak intensities at 3274–3282 cm^−1^, 2884 cm^−1^, and 2935–33 cm^−1^ corresponded to O—H stretching, C—H vibrations in the CH_3_/CH_2_ groups, deformation of C—H in the CH_3_ groups, and aromatic ring vibrations, as illustrated in Fig. 2A. 1644–1650 cm^−1^ bands intensities indicated amide-Ⅰ group, C=O stretching (syringyl-guaiacyl), 1592 cm^−1^ shown amide-ⅠⅠ group, C—H stretching, N—H bending, and 1220–1350 cm^−1^ represented amide ⅠⅠⅠ group, C=O, C=N stretching and N=H bending (Fig. 2A). 1350 cm^−1^ representing the presence of α-helix, suggesting a more accurate protein secondary structure analysis (Khosa et al., 2013). The peaks in all the films at 1035–1032 cm^−1^ specified the presence of cystine S-sulfonated residues in the films (Shavandi et al., 2017). Changes found in the frequency and intensities of absorption bands showed the role of AL and EMAL as reinforcing fillers in the film network.

### Thermal stability analysis of films

#### Thermogravimetric analysis (TGA)

Weight loss and decomposition temperature of the films under continuous heating were observed by TGA and DTG. The thermal behavior TGA and DTG curves of K, AL + K, LML + K, and DML + K films are presented in Fig. 2B-C. Our samples showed almost similar trends of thermal stability by TGA curves. A 3-step decomposition was observed for all film samples, with the first stage of sharp weight loss occurring at 180 °C due to loss of bound water and glycerol. Previous studies have indicated the similar decomposition during thermal stability analysis due the glycerol (Murillo-Morales et al., 2023, Shavandi et al., 2017). The second weight reduction in all film samples (K, AL + K, LML + K, and DLM + K) was recorded at 400–420 °C. It is due to proteins degradation, K disulfide bonds, and lignin C—C bonds breakage (Chen et al., 2021). The third weight reduction in all samples was recorded at 600–700 °C because of thermally decomposed K, AL, and EMAL and the de-crosslinking of these polymers. Our results of TGA indicates that the addition of EMAL resulted in improved thermal stability of the film samples, because of the increase in thermal decomposition and decrease in weight reduction in all three steps of decomposition (Fig. 2B).

According to the DTG curves of the film samples (Fig. 2C), two degradation temperature steps were observed. The first degradation temperature at 107 °C and the second at 253 °C was recorded in the K film sample. Besides, with the addition of AL and EMAL, the degradation temperature in both steps was slightly changed (Fig. 2C). The films AL + K, LML + K, and DML + K indicated that the inclusion of AL and EMAL contributes to an enhancement in the thermal stability of the film samples. A sharp *endo*-thermic peak in the DSC curves of the film samples was found at 120–150 °C (Fig. 2D), indicates the loss of water (Shavandi et al., 2017).

### Scanning electron microscopic (SEM) observation of lignin/keratin films

**Fig. 3:**
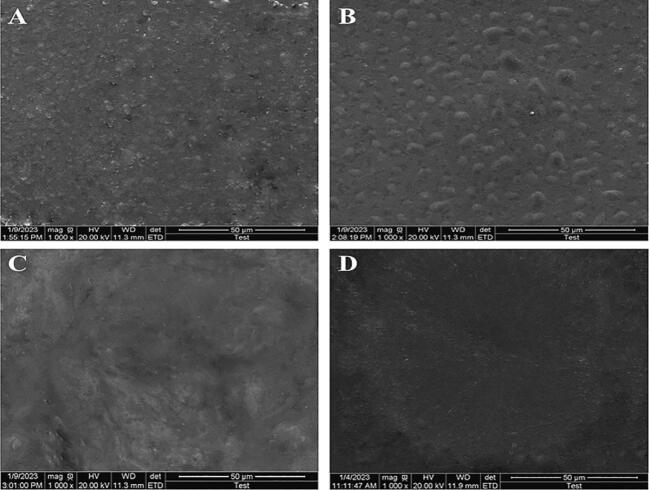
Scanning electron micro-structure of films (A-D). Keratin film (A), alkali lignin film (B), laccase-modified lignin film (C), and DyP-modified lignin film (D).

The scanning electron microscopic observation of AL and EMAL films are displayed in Fig. 3A – D. In Fig. 3A and B, the microstructure of the K film did not appear smooth and homogenous. The addition of AL increased the roughness of film surface (Fig. 3B). AL + K film showed a fluctuating and continuous structure without cavities and holes, which can be attributed to the chemical interaction between K and AL (Fig. 3B). Besides, the addition of EMAL induced physical changes that appeared smooth and homogenous without cavities, edges, and holes, because of the lower molecular weight fractions of EMAL, and short lignin chains after enzymatic treatment leads to better homogenization of the mixture (Fig. 3C and D).

### Surface chemistry via X-ray photoelectron spectroscopy (XPS) of films

**Fig. 4:**
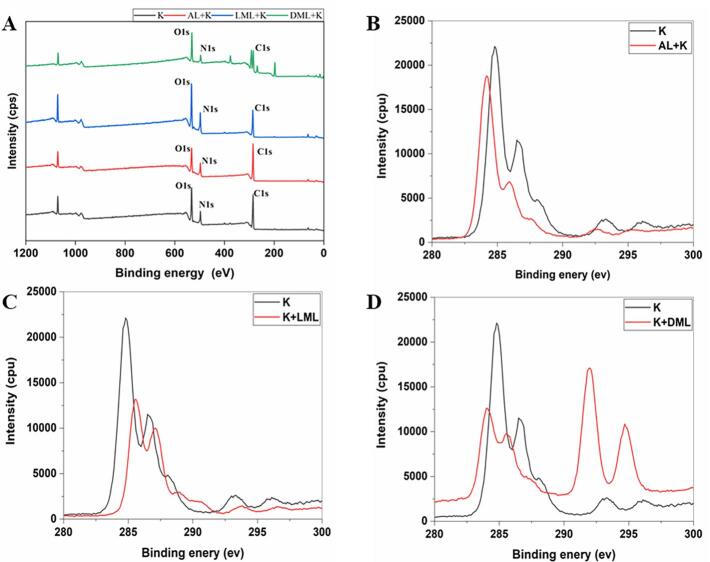
X-ray photoelectron spectroscopy (XPS) analysis of films (A-D). Survey spectrum (A), C 1S peak from XPS spectra of keratin and alkali lignin films (B), C 1S peak from XPS spectra of keratin and laccase modified lignin films (C), and C 1S peak from XPS spectra of keratin and DyP modified lignin films (D). K (keratin), AL (alkali lignin), LML (laccase modified lignin), and DML (DyP modified lignin).

Highly accurate elemental/chemical and surface/physical compositions of materials prepared from biopolymers/macromolecules can be obtained by XPS analysis (Yaseen et al., 2021) XPS covered both physical and chemical changes that occurred on the surfaces of the prepared materials. Herein, XPS analysis of films are shown in Fig. 4A – D. Single peaks of C 1 s, N 1 s, and O 1 s were confirmed by the survey spectrum of XPS (Fig. 4A). Previous studies have described the peaks at 284.7 eV, 285.9 eV, and 288 eV for C—C, C—O—C, and O-C=O, associated with C 1 s spectra (Yaseen et al., 2021). And peaks at 284.5 eV, 286.5 eV, and 288 eV were associated with C—C, C—O/C—N, and C=O. Our results displayed the peaks at 284–284.5 eV, 286–286.5 eV, and 287.5–288 eV representing C—O/C—N and C=O, associated with C 1 s spectra. Three another peaks at binding energies 284–284.5 eV, 286–286.5 eV, and 287.5–288 eV, indicates C—C, C—O—C, C—O/C—N, O-C=O, and C=O in K and AL + K films (Fig. 4B). With the addition of EMAL the peaks were obtained at 285.5 eV, 287 eV, and 289 eV (Fig. 4C). And on the film surface of DMAL + K, two more peaks were observed at 292.5 eV and 295 eV (Fig. 4D). Our results of XPS exhibited two additional peaks, representing C—C/C—H and C=O, might be because of AL and EMAL addition to the film matrix.

### Antibacterial capabilities of lignin’s and keratin films

**Fig. 5:**
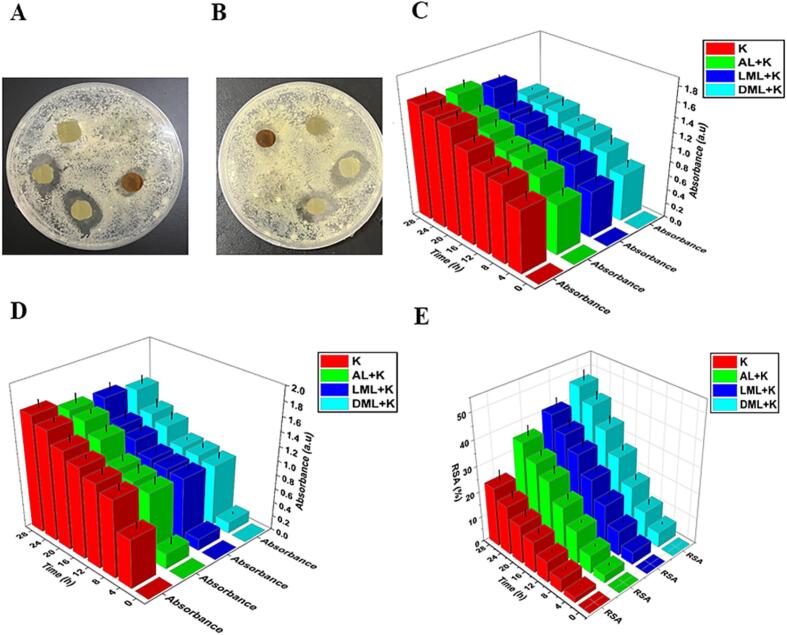
Antibacterial and antioxidant activities. (A-D), antibacterial activity of films against gram-positive *S. aureus* and gram-negative *E. coli* via disc diffusion and optical density methods. Disc diffusion activity of films against *S. aureus* (A) and *E. coli* (B); Optical density activity of films against *S. aureus* (C) and E. coli (D). K (keratin), AL (alkali lignin), LML (laccase modified lignin), and DML (DyP modified lignin). (E), antioxidant activity (radical scavenging activity) of films evaluated by 2,2-diphenyl-1-picrylhydrazyl (DPPH).

The preparation of antimicrobial packing material can surprisingly protect the packed materials from contamination. We used a gram-positive *S. aureus* and a gram-negative *E. coli*, to elucidate the anti-bacterial capabilities of our prepared films by standard broth dilution (optical density) and disc diffusion methods (Fig. 5A – D). Previously, the antibacterial activity of lignin containing composites against *S. aureus* (with a thicker peptidoglycan layer) and *E. coli* (with more fatty acids), concluded that phenolic hydroxyl group of lignin can influence bacterial growth (Pothal et al., 2023, Yun et al., 2021). Our results of the antibacterial activity indicate, effective decrease in the growth of *S. aureus* and *E. coli* strains, by forming a clear zone around the discs containing AL and EMAL (DML: 25–20 mm in length and 15–10 mm in width, LML: 21–16 mm in length and 13–10 mm in width, and AL: 16–11 mm in length and 9–7 mm in width) (Fig. 5A and B). Thus, the films containing AL and EMAL showed bacterial inhibition against both bacterial strains, whereas no growth inhibition zone was observed in the film containing only K. Previous studies indicated similar bacterial growth inhibition against lignin and chitosan bio-composite film (Pothal et al., 2023).

For optical density/cell density, the antibacterial activity of films (K, AL + K, LML + K, and DML + K) was observed using a standard broth diluted medium containing *S. aureus* and *E. coli* strains (Fig. 5C and D). Our results indicate, that the films contain AL and EMAL added into the growth medium of *S. aureus* and *E. coli* strains decreased the cell density/cell growth of the bacterial strains as long as the incubation time was increased. The growth of *S. aureus* and *E. coli* was lower in AL and EMAL samples, as compared to K samples, suggesting that the films with AL and EMAL possessed potent antimicrobial inhibition.

### Antioxidant activity of keratin/alkali and decolorized lignin

The antioxidant capabilities of polymeric packing materials, extends the shelf life of packed materials by protecting them from oxidation (Lai, 2021). To evaluate the antioxidant activity of the developed films, DPPH free RSA method was used to assess the impact of AL and EMAL on antioxidant capacity. Cross-ponding kinetics of the RSA activity are shown in Fig. 5E. The EMAL films, exhibited significantly higher RSA than those of AL and K films (Fig. 5E). Although, the RSA of AL is higher than only K film. Because phenolic hydroxyl groups are the main reason of antioxidant properties of lignin, and lignin are rich in several functional groups including phenolic hydroxyl. However, lignin’s are polymers with high molecular weight, which effect its mobility and reactivity. Depolymerization of lignin following bacteria-derived-enzymes (i.e. Lacc and DyP., etc.) treatment leads to its degradation into smaller aromatic monomers (Chauhan, 2020). Thus, the increased antioxidant activity of EMAL films might be because of enzymatic treatment.

## Conclusion

By co-dissolving water and ethanol, the aromatic biopolymer AL, EMAL and the fibrous protein K were combined to form sustainable films. FT-IR spectrum of films confirmed the hydrogen and aryl-π bonding among the phenoxide anions, aryl hydroxyl, and amide groups of AL, EMAL, and K. The enzymatic treatment not only improved the mechanical strength of the films but also minimized the risk of bacterial infection and oxidation. By focusing on the functional and structural properties, the resulting films exhibited excellent antibacterial, antioxidant, mechanical properties and barrier performances against water permeation and moisture content. The enhanced bacteriostatic effect against *S. aureus* and *E. coli*, higher RSA, enhanced EAB and water barrier properties indicates the increased concentration of phenolic hydroxyl groups and low molecular weight lignin fractions, achieved after enzymatic treatment. Further food and pharmaceutical products packing analysis of the developed EMAL films would explore their potential use in packing applications.

## CRediT authorship contribution statement

**Syed Waqas Ali Shah:** Conceptualization, Methodology. **Keyu Ma:** Visualization, Investigation. **Riaz Ullah:** Validation, Software. **Essam A. Ali:** Funding acquisition. **Abdul Qayum:** Data curation. **Zahoor:** . **Nisar Uddin:** . **Daochen Zhu:** Supervision.

## Declaration of competing interest

The authors declare that they have no known competing financial interests or personal relationships that could have appeared to influence the work reported in this paper.

## Data Availability

Data will be made available on request.
